# Salvage radiotherapy with simultaneous integrated boost in non small-cell lung cancer patients with mediastinal relapse after surgery: a pilot study

**DOI:** 10.1186/s13014-018-1155-2

**Published:** 2018-10-23

**Authors:** L. Nicosia, L. Agolli, C. Reverberi, V. De Sanctis, L. Marinelli, G. Minniti, J. Di Muzio, M. Valeriani, M. F. Osti

**Affiliations:** 1 Department of Radiation Oncology, Sant’Andrea Hospital, “Sapienza” University of Rome, Via di Grottarossa 1035-1039, 00189 Rome, Italy; 2 Department of Radiation Oncology, Faculty of Medicine, University Hospital Carl Gustav Carus, Technische Universität Dresden, Dresden, Germany; 3 Radiation Oncology Unit, UPMC Hillman Cancer Center, San Pietro Hospital, Rome, Italy; 4 IRCCS Neuromed, Pozzilli (IS), Italy; 5 Department of Oncology, Radiation Oncology, University of Torino, Torino, Italy

**Keywords:** Mediastinal recurrent NSCLC, Post-surgical relapse, Salvage radiotherapy, Intensity modulated radiotherapy with simultaneous integrated boost

## Abstract

**Background:**

The aim of our study was to evaluate feasibility, toxicity profile and local control of salvage intensity modulated radiotherapy (IMRT) delivered with simultaneous integrated boost (SIB) associated or not to concomitant weekly cisplatin in patients affected by NSCLC with mediastinal nodal recurrence after surgery. Patterns of recurrence, outcomes and prognostic factors were assessed.

**Methods:**

Fourteen consecutive patients received 25 fractions of 50Gy/2Gy to the elective nodal stations and boost up to 62.5Gy/2.5Gy to the macroscopic lymph node metastases. Concomitant weekly cisplatin (40 mg/m^2^) was administered to 8 (57.1%) patients.

**Results:**

Five (35.7%) patients experienced grade 2 pneumonitis and 5 (35.7%) patients had grade 2 esophagitis. One case of grade 3 pneumonitis occurred and was successfully treated with antibiotics and steroids with no sequelae. No patient recurred locally in the boost volume (local control 100%). Loco-regional control was 79% with 3 patients that developed nodal recurrence principally marginal to the elective volume. Seven patients developed distant metastases. Median PFS was 7 months. The nodal involvement of station 7 was associated to a significantly lower median metastasis-free survival (4 months vs. not reached, *p* = 0.036).

**Conclusions:**

Salvage radiotherapy with IMRT-SIB is a feasible and a well-tolerated treatment option for mediastinal recurrent NSCLC after surgery. The role of more intensified radiation regimens and association to systemic therapy remain to be evaluated in larger cohorts.

## Background

Surgery is the gold standard in the treatment of resectable stage I-IIIA non small-cell lung cancer (NSCLC) with 5-years survival rates of 92% for early stage and 26% for locally advanced disease, respectively [1]. Relapse after surgery may occur in up to 50% of patients depending on the pathological stage [2] and recurrence occurs mainly in the mediastinal lymph nodes in 8–37% of cases [3, 4]. Recent data showed that the routine use of post-operative radiotherapy (PORT) after complete resection has a negative effect on survival [5], therefore, the role of mediastinal irradiation can be evaluated in the case of loco-regional relapse.

Treatment options in of mediastinal recurrence could be surgery, chemotherapy, radiotherapy (RT), or a combination. Nevertheless, the best approach remains uncertain. Some studies reported long-term survival and in some cases even cure after intensive local treatment in a selected population of patients with mediastinal lymph node recurrence after surgery and no distant metastases [6–8].

Systemic therapy alone is necessary, but not very efficient to contain the macroscopical active sites of disease. An aggressive local therapy such as surgery or high-dose RT is required. The reported 5-years survival in patients with loco-regional relapse/persistence treated with surgery ranged from 13 to 25% [9, 10]. Unfortunately, in patients already resected a second surgery is not always feasible. This category of patients often presents with related-symptoms and associated comorbidities; RT could represent a valid alternative comparable to surgery in terms of survival and with acceptable toxicity rates [11, 12]. Most of the studies evaluating this topic are limited from the inhomogeneous treatment schedules and the use of old irradiation techniques. The advantage of modern techniques is to escalate the dose to the tumor and improve toxicity profile in order to obtain good tumor control and better tolerance rates.

We reported feasibility, safety and local control of NSCLC patients presenting post-operative mediastinal nodal recurrence, who received salvage intensity modulated radiotherapy - simultaneous integrated boost (IMRT-SIB) with curative intent.

## Methods

We retrospectively reviewed a series of patients with NSCLC and post-operative mediastinal nodal relapse, treated with salvage IMRT-SIB associated or not to concomitant chemotherapy.

Pre-treatment evaluation included: physical examination, total body computed tomography (CT) scan and 18-fluordeoxyglucose positron emission/ CT (18FDG-PET/CT), both not older than 1 month, lung function test, total blood count and kidney function test.

### Treatment characteristics and planning

All patients underwent pretreatment planning CT in the supine position using a wing-board system for immobilization. Planning CT images were matched with contrast medium diagnostic CT and 18FDG-PET/CT images using automatic matching for the target volume delineation. Gross Tumor Volume (GTV) encompassed the positive mediastinal lymph nodes (PET positive and/or ≥ 10 mm). The elective clinical target volume (CTV-elective) encompassed the nodal stations based on primary tumor location: stations 2R, 4R, 7 and 10R for tumors in the superior right lobe (SRL) and middle lobe (ML); stations 4R, 7, 10R for tumors of the inferior right lobe (IRL): stations 2 L, 4 L, 5, 6, 7, 10 L for tumors in the superior left lobe (SLL); and stations: 4 L, 5, 6, 7, 10 L for tumors located in the inferior left lobe (ILL). Afterwards, GTV and CTV were expanded 5 mm and 7 mm in all directions to generate planning target volume 1 (PTV1) and PTV2, respectively.

All patients were treated with IMRT-SIB technique using multiple coplanar and non-coplanar fields. The margins were maintained small in order to avoid potential toxicities and an image guidance with kilovolt on-board cone beam CT was performed daily in order to guarantee an adequate positioning of the patients. The images from the cone-beam CT were matched to the planning CT prior to each treatment based on the initial automatic bone alignment, followed by a soft tissue/target alignment. Radiation therapy was delivered by a linear accelerator using 6-MV photon beams. The radiation treatment regimen consisted of 50Gy in 25 fractions of 2Gy each to the PTV2, with a simultaneous integrated boost of 62.5Gy in 25 fractions of 2.5Gy each on the PTV1.

The BED (biological equivalent dose 10 [BED10]) at the PTV1 was 78Gy. The dose was prescribed at the 95% isodose with normalization to the maximal dose. The treatment was administered 5 times per week for 5 weeks. Dose constraints for critical normal structures are reported in Table 1. Concomitant chemotherapy consisted in weekly cisplatin (40 mg/m^2^), given on days 1, 6, 11, 16, 21. Chemotherapy was not administered in cases of inadequate renal function or refusal by the patients.

**Table 1:** Dosimetry and dose constraints to critical normal structures

PTV1	Median 45.8 Gy (41.8–48.2Gy)
PTV2	Median 59.8 Gy (57.3–60.1Gy)
Mean Lung Dose	Median 10.3 Gy (6–15.3Gy)
Bilateral lung V20	Median 16.45% (7.6–33.1%)
Ipsilateral Lung V5	Median 62.45% (39.9–81.1%)
Ipsilateral Lung V20	Median 28% (17.9–42.5%)
Controlateral Lung V5	Median 45.75% (2.3–69.1%)
Controlateral lung V20	Median 16.35% (0.6–48%)
Heart V5	20.2% (7.1–66.1%)
Heart V30	2.75% (0–12.3%)
Esophagus V11.5	36% (21.1–68.5%)
Esophagus V18	45.15% (27.3–69.1%)
Esophagus V27	52.35% (26.9–69.8%)

### Follow-up and statistics

All patients underwent weekly clinical evaluations and routine blood examinations during radiation therapy. Treatment-related toxicities were graded according to the CTCAE v 4.0 scale. Follow-up was performed every 3 months for the first 2 years after RT and then every 6 months afterward. A total body CT with contrast medium was performed at 1 month after RT and every 6 months afterward. A post treatment 18FDG-PET/CT was performed at 3 months after RT and if there was suspicion for tumor recurrence/progression at CT images. The local/regional recurrence was based on new evidence or marginal regrowth of the disease on follow-up CT scans and higher uptake on the 18FDG-PET/CT.

Statistical analysis was performed using SPSS, version 16.0, software (SPSS, Inc., Chicago, IL). Primary end-points were toxicity and local control. Secondary end-points were pattern of failure, survivals and possible prognostic factors. Local progression-free survival (LPFS) was defined as the time to in-field or marginal regrowth of the disease or death. PFS was defined as the time to any progression or death. Metastasis-free survival (MFS) was defined as any site of distant progression (including the lungs) or death. Survivals were calculated from the date of initial RT and were estimated using the Kaplan- Meier method. Clinical prognostic factors such as age, sex, histological subtype, number and station of involved lymph nodes, PTV size, previous chemotherapy, and type of response were included in the statistical analysis. Univariate analysis was performed to determine significant prognostic factors using the log-rank test. A *p*-value < .05 was considered statistically significant.

## Results

### Patients’ characteristics

From January 2014 to September 2016, 14 consecutive patients with NSCLC and post-operative mediastinal nodal relapse were treated with salvage IMRT-SIB associated or not to concomitant chemotherapy.

Median age was 68 years (range, 51–78 years). Eleven (78.5%) patients were male, 3 (21.5%) were female. Patients had ECOG (Eastern Cooperative Oncology Group Criteria) ≤2 and received no previous thoracic RT. All patients underwent surgery and mediastinal lymphadenectomy before salvage RT. Five (35.7%) patients received pre-operative chemotherapy with platinum-based regimen and 9 (34.3%) patients received subsequently platinum-based adjuvant chemotherapy. The median interval between primary surgery and mediastinal recurrence was 10 months (range, 2–18 months). Patient’s characteristics are summarized in Table 2.

**Table 2:** Patients’ characteristics (*n* = 14)

Age (median, years)	68
Range (years)	51–78
Sex	
• Male	11 (78.5)
• Female	3 (21.5)
Histology	
• Adenocarcinoma	12 (85.6)
• Squamocellular	2 (14.4)
Pathological Stage	
• IB	2 (14.4)
• IIA	6 (42.8)
• IIIA	6 (42.8)
Neoadjuvant Chemotherapy	
• Platinum-based	5 (35.7)
• No	9 (64.3)
Initial surgery	
• Lobectomy + LAD	13 (92.8)
• Atypical resection + LAD	1 (7.2)
Adjuvant Chemotherapy	
• Platinum-based	8 (57.2)
• Vinorelbine monotherapy	1 (7.1)
• No	5 (35.7)
N° of pathological nodal stations involved at diagnosis	
0	2 (14.3)
1	6 (42.8)
2	3 (21.5)
3	1 (7.1)
4	2 (14.3)

LAD: lymphadenectomy

### Local control and toxicity profile

Five (35.7%) patients experienced grade 2 pneumonitis and 5 (35.7%) patients had grade 2 esophagitis. One patient developed grade 1 leucopenia and grade 2 thrombocytopenia; radiochemotherapy was prudently interrupted for 5 days until achievement of normal blood count and was subsequently continued with no other interruption or toxicity. No treatment-related deaths occurred. One (7.1%) case of G3 pneumonitis occurred and was then successfully treated with antibiotics and steroids with no sequelae. The patient had a known diagnosis of chronic obstructive pulmonary disease (COPD) and a probable reactivation of the disease could be hypothesized, also considering the retained dose constraints for the lung (V20 29.9%, MLD 15.1%). See Table 3.

**Table 3:** Acute and late toxicity (sec. CTCAE 4.0)

	G1–2	G3	G4–5
Acute toxicity			
Pneumonitis	5 (35.7)	1 (7.1)	0
Esophagitis	5 (35.7)	0	0
Nausea	3 (21.5)	0	0
Leukopenia	1 (7.1)	0	0
Thrombocytopenia	1 (7.1)	0	0
Fatigue	2 (14.3)	0	0
Skin erithema	1 (7.1)	0	0
Late toxicity			
Fibrosis	4 (28.6)	0	0

Five (35.7%) patients did not receive concomitant chemotherapy for inadequate kidney function or refusal. No patient recurred locally in the boost volume (local control 100%). Loco-regional control was 79% with three patients that developed nodal recurrence principally marginal to the elective volume.

### Patterns of relapse, survival and prognostic factors

After a median follow up of 12 months (range: 3–28 months), 3 patients developed regional failure: one patient had lymph node metastases in station 7 and 5 marginal to the elective volume; 2 patients developed lymph node metastasis in station 7: in the elective irradiated volume and marginal to the elective volume, respectively. Seven patients developed distant progression: 2 patients had solitary brain metastases, one patient had diffuse lung and nodal metastases, one patient developed diffuse liver metastases, one patient had bone and brain metastases and one patient had bone metastases. See patterns of relapse in Table 4.

**Table 4:** Patterns of recurrence and treatment

Patient	Age	Site of Boost Volume	CCT	Dosis SIB (Gy)	Volume/Volume SIB (cc)	Local relapse (boost)	Nodal regional relapse	Distant recurrence	Therapy after recurrence
1	78	2 L, 4 L, 5, 6	Yes	50/62.5	323.24/80	No	No	Lung, nodes	BSC
2	55	4R, 10R	Yes	50/62.5	166.48/52.43	No	7	Brain, bone	Palliative RT
3	67	4R, 5, 10R	No	50/62.5	213.81/14.91	No	No		
4	78	rT, 10R	No	50/62.5	201.06/11.35	No	7 and 5		BSC
5	75	2R, 4R, 5	Yes	50/62.5	402.31/98.14	No	No		
6	69	5, 6, 10 L	No	50/62.5	203.58/76.48	No	No		
7	56	10R	Yes	50/62.5	64.16/5.42	No	No		
8	55	2 L, 4R, 6	Yes	50/62.5	148.86/10.29	No	No		
9	51	2R, 4R, 5, 7, 10R	No	50/62.5	313.01/193.46	No	No		
10	57	2R, 4R, 5, 7, 10R	No	50/62.5	408.43/95	No	No	Brain	SRS 9 Gyx3
11	77	7	No	50/62.5	289.92/34.99	No	No	Liver	BSC
12	58	4R	Yes	50/62.5	141.27/95.95	No	No	Brain	3 SRS 20 Gy
13	69	4R, 10R	Yes	50/62.5	121.91/50.37	No	7	Bone	CT
14	69	4R	Yes	50/62.5	134.19/19.86	No	No	Lung, nodes	BSC

*CCT*: concomitant chemotherapy, *SIB*: simultaneous integrated boost, *CT*: chemotherapy, *RT*: radiotherapy, *BSC*: best supportive care, *SRS*: stereotactic radiosurgery

At the time of the analysis, 6 (42.8%) patients were deceased: 4 of them due to disease progression, 2 of them had a worsening of the known COPD and one died of massive thrombosis. Median PFS was 7 months (range: 3–28 months) and 1-year PFS was 39.7%. Median LPFS was 26 months (range: 3–28 months) and 1-year LPFS was 80%. Median MFS was 9 months (range: 3–28 months) and 1 year MFS was 46.3%.

Concomitant chemotherapy did not significantly predict for a longer LPFS, PFS or MFS. The nodal involvement of station 7 was associated to a significantly lower median MFS (4 months vs. not reached, *p* = 0.036). No other significant factors were found.

## Discussion

This is a pilot study of patients affected by NSCLC presenting with post-operative mediastinal lymph node relapse and treated with IMRT-SIB technique associated or not to weekly cisplatin. The purpose of the study was to establish the safety and feasibility of the hypofractionated RT schema. Our results are in accordance to those presented by other authors. Bae et al. [12] treated 64 patients between 1994 and 2007, with a median BED_10_ of 70.2 Gy (range 51.5–85.8 Gy) delivered with various fractionations and concomitant chemotherapy in 21.9% patients. Toxicity profile was characterized by one (1.6%) case of grade 4 pneumonitis and 6 (9.4%) cases of grade3 pneumonitis. Kim et al. [13] reported 5.3% and 3.5% rates of grade 3 pneumonitis and esophagitis, respectively, in 57 NSCLC patients with loco-regional relapse after surgery treated with different RT schedules (median BED_10_ 79.2 Gy; range 58.5–84 Gy) and concomitant chemotherapy (73.7% of patients).

Our treatment schedule was well tolerated, with only one case of grade 3 pneumonitis in a patient with known COPD. The use of IMRT in our population can explain the relatively low rate of severe toxicity, as compared to the higher rates (2.6–65.5%) of previous studies, where 3D-CRT, techniques or more toxic chemotherapeutic regimens were used. A summary of previous studies showing the toxicity profile, type of treatment and RT regimens is reported in Table 5.

**Table 5:** Summary of literature on toxicity and relative doses in salvage radiotherapy for locoregional recurrent NSCLC after surgery

Author	Type of study	N° of patients	Time period	Median FUP	RT technique	Treatment volume	Median Dose (Gy)	Concurrent chemotherapy (n°)	Toxicity (%)
Kagami et al., 1998 [20]	Retrospective	32	1981–1991	n.s.	AP-PA until 40 Gy, then opposite oblique technique	PTV: GTV (margins n.s.)	47.5–65	–	No G3
Uno et al., 2005 [21]	Retrospective	21	2000–2004	2–8 months	3D-CRT	PTV1: uninvolved mediastinal and ipsilateral hilar lymphnodes+ 10–15 mm PTV2:GTV + 10 mm	60 (46–60)	wCBDCA (7) wCBDCA-PTX (2) wCBDCA-VDS (1) wCBDCA-VNB (1) wCDDP (1)	Pneumonitis G3 (4.7) Hematologic G3 (4.7) Hematologic G4 (4.7)
Kelsey et al., 2006 [22]	Retrospective	29	1991–2003	n.s.	n.s.	PTV: ENI (27/29; n.s. in others)	66 (46–74)	NAD to RT (7) Concomitant (8)	Esophagitis + stenosis G3 (6.8)
Bae et al., 2012	Retrospective	64	1994–2007	32 months	3D-CRT	PTV: GTV + 10–20 mm	Median BED_10_ 70.2 (51.5–85.8)	wCDDP-PTX (12) CDDP-Eto q28 (2)	Pneumonitis G3 (9.4%) Pneumonitis G4 (1.6%)
Bar et al., 2013 [18]	Retrospective	30	1999–2007	n.s.	3D-CRT (43)	n.s.	63.5 (26–66)	CDDP-VNL q21 (14) wCBDCA-PTX (7) CDDP-Eto q 21 (4) Other platinum reg. (5)	Pneumonitis G3 (7) Pneumonitis G4 (3.5) Esophagitis G3 (14) Febrile neutropenia (14) Esophageal stenosis (7) Other G3/4 (5/1): hearing loss (2), depression (1), neuropathy (1), nausea (1), pulmonary embolus (1)
Lee et al., 2013 [14]	Retrospective	38	2001–2009	26.4 months	3D-CRT	PTV: GTV + 10 mm for the CTV + 5–15 mm Ipsilateral hilum usually included,	Median BED_10_ 74.4 (58.5–97.5)	CDDP-Eto (9) CBDCA-PTX (3)	Esophagitis G3 (2.6%)
Takenada et al., 2015	Retrospective	35	2000–2011	n.s.	n.s.	n.s.	60 (30–60)	CDDP-S1 (11) CDDP-Tegafur-Uracil (8) CBDCA-VNL (10) Other CDDP reg. (4) Other CBDCA reg. (2)	Esophagitis G3 (9) Neutropenia G3 (14) Neutropenia G4 (20) Cardiac G3 (2) Miscellaneous infection (9) Other G3 (11)
Kim et al., 2017 [13]	Retrospective	57	2004–2014	53.6 months	3D-CRT	PTV: GTV + 5–8 mm for the CTV + 10 mm	Median BED_10_ 79.2 (58.5–84)	wCDDP-PTX (40) CDDP-Eto q21 (2)	Pneumonitis G3 (5.3) Esophagitis G3 (3.5)
Nakamichi et al., 2017 [19]	Retrospective	74	2000–2010	n.s.	n.s.	Lung lesions+recurrent lymphnodes+regional nodes	Median 60 (50–70)	CDDP-VNL q28 (14) CBDCA-PTX (3) CDDP-PTX (1)	RT group (56): Pneumonitis G3 (5) Late pulm. Toxicity (2) Other non-hematologic (2) R-CT group (18): Febrile neutropenia (17) Pneumonitis G3 (5) Esophagitis Ge (5) Other non-hematologic (5)
Seol et al., 2017 [16]	Retrospective	31	2008–2013	14 months	3D-CRT	PTV: GTV + 10–20 mm + adjacent nodal areas (mediastinal and hilar) considered at risk+ 3–5 mm	66 (51–66)	NAD CT (7) Concomitant CT (7) NAD CT + R-CT (1) RT + Adj CT (1)	Pneumonitis G3 (3.2)
Nicosia et al. (present study)	Retrospective	14	2014–2016	12 months	IMRT-SIB	PTV1: GTV + 5 mm PTV2: involved nodal station + adjacent stations and hilum + 7 mm	50 + boost 62.5	wCDDP (9)	Pneumonitis G3 (7.1)

*NSCLC*: non-small cell lung cancer; *FUP*: follow-up; *CT*: chemotherapy; *AP-PA*: antero-postero parallel opposite technique; 3D-CRT: 3-dimensional conformal radiotherapy; *NAD*: neoadiuvant; *BED*: biological equivalent dose; *R-CT*: radio-chemotherapy; *Adj:* adjuvant; *CBDCA*: carboplatin; *PTX:* paclitaxel; *VDS:* vindesine; *VNB*: vinorelbine; *CDDP*: cisplatin*; Eto*: etoposide

The rationale of treating NSCLC patients with mediastinal nodal relapse retain in the oligometastatic concept: patients with limited metastatic disease may benefit from more aggressive local treatment to the macroscopical disease. Recent series suggest that survival of loco-regional recurrent NSCLC is more similar to that of stage III NSCLC than to stage IV. Therefore, it is conceivable that a dose of RT >66Gy and the addition of systemic therapy may improve local control having a favorable effect on survival [1, 14–17].

Bar et al. [18] treated 30 patients with different RT schedules (median dose 63.5 Gy) and platinum-based chemotherapy obtaining a response rate of 70% and 2- and 3-years overall survival (OS) of 50.8% and 38.7%, respectively. Nakamichi et al. [19] reported a series of 74 patients treated with RT alone 60 Gy (56 patients) or RT 60 Gy associated to concomitant chemotherapy (18 patients). The response rate after RT alone and combined therapy were 68% and 78%, respectively, and the use of the concomitant chemotherapy predicted for better OS and PFS. The median OS for RT alone and radiochemotherapy patients were 33.1 and 79.6 months, respectively, and the 5-years OS were 35% and 53%, respectively. Kim et al. [13] reported rates of 2-years LRFS and OS of 70.9% and 62.4%, respectively. Treatment failure globally occurred in 68.4% of patients with 8.8% of isolated in-field failure.

Local control of our series was in line with previous studies with 50% of complete response at 1-year, median LPFS of 26 months and 1- and 2-years LPFS of 80% and 40%, respectively. In-field recurrence occurred all within the 50 Gy volume, so that the dose in the boost volume seems adequate to control the macroscopical disease, even if a longer follow-up would be required.

The contribution of metastatic spread was difficult to assess in this series, also considering that the analysis of survival was beyond the intentions of this pilot study. Metastatic spread occurred in 50% of patients after a median of 9 months. This could be in accordance with two hypothesis: first of all, it was a disseminated disease from the beginning, and second of all, there was poor efficacy of the concomitant chemotherapy. We are more likely to consider the second option as more plausible, since we accurately staged all the patients before treatment with CT scan and 18FDG-PET/CT. Moreover, the systemic control of previous studies, where regimens more intensive were administered, are consistent with this hypothesis.

To our knowledge this is the first study that reported the use of IMRT-SIB in a population of mediastinal nodal recurrent NSCLC. Our preliminary data, taking into account the retrospective nature of the study, showed optimal tolerance and promising local control. A phase II study with a larger population is mandatory to assess the effect of a more intensive concomitant chemotherapy regimen that could synergize with the proposed fractionation delivered with modern and precise techniques, with the aim to improve survival and minimize side effects.

## Conclusions

Aggressive local therapy associated to systemic therapy could be a good option in the treatment of postoperative mediastinal nodal relapse in NSCLC patients unfit to receive a second surgery. In this particular population of oligometastatic NSCLC patients, a modern radiotherapy delivered with accurate techniques and a combined systemic approach may lead to good outcome and tolerability. Better distant control might be obtained with more intensified systemic therapy and should be prospectively evaluated in a larger series.

## Abbreviation

18FDG-PET: 18fluorodeoxyglucose-positron emission tomography
3D-CRT: 3-dimensional conformal radiotherapy
Adj: Adjuvant
AP-PA: Antero-postero parallel opposite technique
BED: Biological equivalent dose
BSC: Best supportive care
CBDCA: Carboplatin
CCT: Concomitant chemotherapy
CDDP: Cisplatin
COPD: Chronic obstructive pulmonary disease
CR: Complete response
CT: Computed tomography
CTCAE: Common Terminology Criteria for Adverse Events
CTV: Clinical target volume
Eto: Etoposid
FUP: Follow-up
GTV: Gross target volume
ILL: Inferior left lobe
IMRT: Intensity-modulated radiation therapy
IRL: Inferior right lobe
LPFS: Local progression-free survival
MFS: Metastasis-free survival
ML: Middle lobe
MLD: Mean lung dose
NAD: Neoadiuvant
NSCLC: Non-small cell lung cancer
OS: Overall survival
PFS: Progression-free survival
PORT: Post-operative radiation therapy
PTV: Planning target volume
PTX: Paclitaxel
R-CT: Radio-chemotherapy
RT: Radiotherapy
SIB: Simultaneous integrated boost
SLL: Superior left lobe
SRL: Superior right lobe
SRS: Stereotactic radiosurgery
VDS: Vindesine
VNB: Vinorelbine
